# Investigating the dislocation reactions on Σ3{111} twin boundary during deformation twin nucleation process in an ultrafine-grained high-manganese steel

**DOI:** 10.1038/s41598-021-98875-z

**Published:** 2021-09-29

**Authors:** Chang-Yu Hung, Tomotsugu Shimokawa, Yu Bai, Nobuhiro Tsuji, Mitsuhiro Murayama

**Affiliations:** 1 Department of Materials Science and Engineering, Virginia Tech, Blacksburg, VA 24061 USA; 2 Faculty of Mechanical Engineering, Kanazawa University, Kanazawa, Ishikawa 920-1192 Japan; 3 Department of Materials Science and Engineering, Kyoto University, Yoshida-honmachi, Sakyo-ku, Kyoto, 606-8501 Japan; 4 Elements Strategy Initiative for Structural Materials, Kyoto University, Yoshida-honmachi, Sakyo-ku, Kyoto, 606-8501 Japan; 5 Institute for Materials Chemistry and Engineering, Kyushu University, Kasuga, Fukuoka 816-8580 Japan; 6 Present Address: School of Materials Science and Engineering, Dalian University of Technology, Dalian, 116024 China

**Keywords:** Metals and alloys, Transmission electron microscopy

## Abstract

Some of ultrafine-grained (UFG) metals including UFG twinning induced plasticity (TWIP) steels have been found to overcome the paradox of strength and ductility in metals benefiting from their unique deformation modes. Here, this study provides insights into the atomistic process of deformation twin nucleation at Σ3{111} twin boundaries, the dominant type of grain boundary in this UFG high manganese TWIP steel. In response to the applied tensile stresses, grain boundary sliding takes place which changes the structure of coherent Σ3{111} twin boundary from atomistically smooth to partly defective. High resolution transmission electron microscopy demonstrates that the formation of disconnection on Σ3{111} twin boundaries is associated with the motion of Shockley partial dislocations on the boundaries. The twin boundary disconnections act as preferential nucleation sites for deformation twin that is a characteristic difference from the coarse-grained counterpart, and is likely correlated with the lethargy of grain interior dislocation activities, frequently seen in UFG metals. The deformation twin nucleation behavior will be discussed based on in-situ TEM deformation experiments and nanoscale strain distribution analyses results.

## Introduction

Ultrafine-grained (UFG) and nanocrystalline metals and alloys have attracted intensive research interest since their birth because of their potential to achieve extraordinary properties over conventional coarse-grained (CG) counterparts. This has led to a series of studies regarding the synthesis, processing, characterization, and potential applications of UFG and nanocrystalline metals and alloys in the past few decades^1–5^. Similar to many other high strength metals and alloys, UFG metals and alloys commonly exhibit a high strength/hardness that is generally accompanied by poor ductility, known as the strength-ductility paradox, regardless of their crystal structure, for example, UFG aluminum (face-centered cubic)^2,6^, UFG iron and interstitial free steels (body-centered cubic)^6,7^, and nanograined copper alloys (face-centered cubic)^2^. The yield strength of materials increases monotonously with decreasing in the grain size, which is generally described by the Hall–Petch relationship^8,9^. The tensile ductility drops immediately when the average grain size becomes smaller than 1 µm, which is related to the characteristics of UFG microstructure, that is, fine grains leave very little space for dislocation dynamics leading to a less enhanced strain-hardening rate and resulting in the plastic instability during deformation^10,11^.

Recently, a high strength and moderate ductility are simultaneously achieved in a UFG magnesium alloy^12^, a UFG Fe–Ni–C metastable austenitic steel^13^, and UFG high manganese Twinning Induced Plasticity (TWIP) steels^14–16^. Experimental results of these materials indicate that nonconventional or reverse-order deformation modes were activated in addition to the normal dislocation slip, specifically, <c + a> dislocations activation in the UFG Mg alloy, martensitic transformation in the UFG metastable austenitic steel, and deformation twinning in the UFG TWIP steels. Tsuji et al.^13^ suggest that the sequential activation of different deformation modes would foster the regeneration of strain-hardening ability during plastic deformation and lead to a high strength and large ductility because of possible interactions between different deformation modes, in other words, overcoming the paradox of strength and ductility. However, the detailed mechanisms to activate such nonconventional deformation modes are not fully understood yet.

The authors have recently investigated the activation factors of different deformation modes in a UFG austenitic TWIP steel^14^. Our results indicate that the activation takes place in the very early stage of plastic deformation like near the macroscopic yield point, and the grain size plays a major role in deformation mode alternation; deformation twin nucleation occurred at the grain boundaries in under-1 µm austenitic grains, while the normal in-grain slip was mostly observed in over-1 µm grains. This grain size dependence could be attributed to the lack of initial mobile dislocations and inactive in-grain dislocation sources; both are characteristics of the UFG microstructure. Because of its technical and scientific importance, the deformation twinning behavior in conjunction with mechanical properties has been extensively studied in conventional CG TWIP steels^17–19^. However, theses deformation twinning mechanisms based on the arrangement of highly coordinated Shockley partial dislocations on {111} slip planes^20–24^ appear to be insufficient to explain the grain boundary mediated deformation twinning in the UFG TWIP steels because the deformation twinning in UFG TWIP steels appears to be highly correlated with grain boundary structure.

Regarding the correlation between grain boundary character and deformation twin mechanism, only handful studies have carried out even on the conventional CG TWIP steels^25,26^. Our recent in-situ TEM deformation study has demonstrated that the deformation twin nucleation at a Σ3{111} twin boundary in the CG microstructure occurs only when a localized stress concentration field formed by dislocation pile-up at the grain boundary^26^. This deformation twin nucleation behavior is unlikely feasible in the UFG microstructure because the grain interior dislocation activities are no longer predominant thus limiting in-grain dislocation pile-up at a twin boundary ^14,27^. This leads an open question, that is, what mechanism would promote the deformation twin nucleation at a Σ3{111} twin boundary, which is the dominant boundary type in the UFG TWIP steels, when the localized stress concentration field cannot be generated by dislocation pile-up, or localized stress concentration field is no longer necessary for the deformation twin nucleation in the UFG TWIP steels.

In this study, we aim to address the question by carefully observing the internal behavior of Σ3{111} twin boundaries subjected to external tensile stresses and revealing the deformation twin nucleation process on the boundaries in a UFG high manganese TWIP steel. A carbon-free UFG high-manganese steel was selected for this study to minimize possible additional effects such as the serrated flow that frequently takes place in carbon-containing TWIP steels^28^. The detailed deformed microstructure near Σ3{111} twin boundaries and their local strain level were investigated using transmission electron microscopy (TEM) and scanning transmission microscopy (STEM) techniques to shed a light on the atomistic process of deformation twin nucleation in the UFG microstructure.

## Results

**Figure 1:**
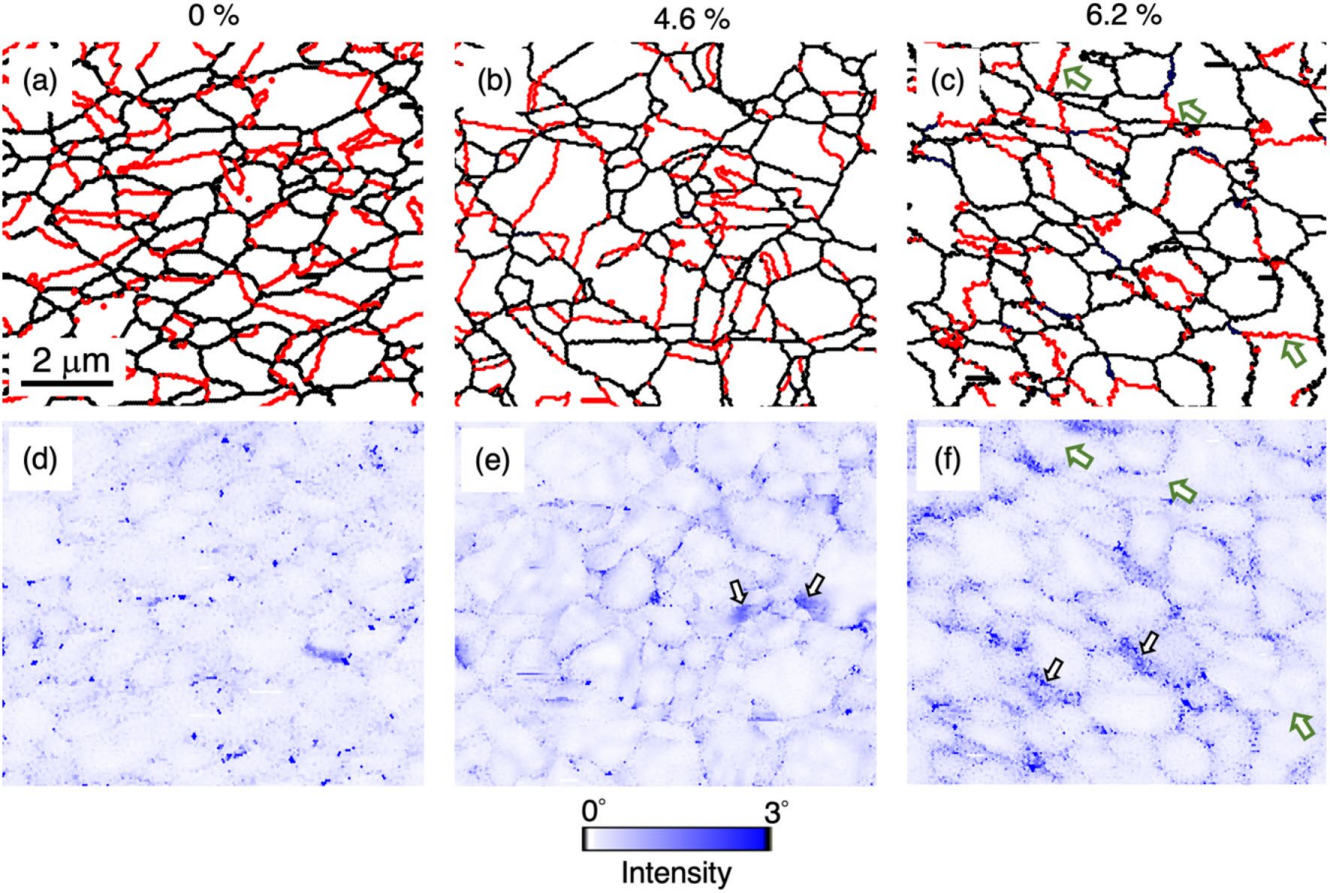
EBSD maps of undeformed (0%) and two tensile strained (4.6% and 6.2%) UFG samples with the scan step size of 50 nm: (**a**–**c**) grain boundary maps, (**d**–**f**) kernel average misorientation maps of (a-c). Black lines in (**a**–**c**) represent the high angle boundaries with rotation angle (θ), 15° ≤ θ < 60°, and red lines represent Σ3{111} twin boundaries. The average misorientation showing in (**d–f**) was calculated by taking the points as well as all of its nearest neighbors into account with a criterion that the misorientation exceeding the threshold (3°) was discarded from the calculation. The stress concentration and several representative Σ3{111} twin boundaries are indicated by white arrows and green arrows, respectively.

The microstructure of both undeformed and tensile-strained UFG TWIP steel samples were firstly characterized by EBSD. The UFG TWIP steel has the identical chemical composition and stacking fault energy (SFE) with the CG TWIP steel we previously studied (Fe-31Mn-3Al-3Si wt.%, SFE = 40 mJ m^−2^)^29^, while the average grain size is about 20 times smaller than that of the CG one, 0.79 µm instead of 15.4 µm. Three grain boundary maps for the undeformed and two different tensile-strained samples are shown in Fig. 1a–c, respectively. Black lines represent the high-angle grain boundaries with the rotation angle θ, 15° ≤ θ < 60°, and red lines represent annealing twin boundaries, known as Σ3{111} twin boundaries. Figure 1a demonstrates that the microstructure prior to tensile straining were composed of undeformed single austenite phase.

Kernel average misorientation (KAM) maps for these three samples are shown in Fig. 1d–f. The KAM analysis provides a qualitative analysis of the local misorientation that is strongly influenced by the density of geometrically necessary dislocations (GNDs)^30^. The undeformed sample in Fig. 1d shows very little local misorientation over the entire scanned area containing both in the grain interior and near grain boundary regions, suggesting that only uninfluential amounts of deformations were introduced during the EBSD sample preparation. When the samples were tensile strained to the engineering strain of 0.046 and 0.062, the local misorientation were observed mainly around grain boundaries as indicated by white arrows in Fig. 1e and f. Since the local misorientation is a function of the GND density, the uneven distribution suggests that the heterogeneous dislocation dynamics at individual grain boundaries were somehow triggered then proceeded by the applied tensile stress. Although the level of plastic strain accumulated near grain boundaries cannot be quantified, the level of the plastic strain accumulation appears to be correlated with the grain boundary misorientation. For example, in Fig. 1c and f, the GND density near coherent Σ3{111} twin boundaries (marked by green arrows) are clearly less pronounced than those in other general high-angle grain boundaries. Whether the Σ3{111} twin boundaries were deformed or not cannot be immediately confirmed in here solely based on the EBSD with the scan step size of 50 nm. Therefore, further microstructure observations in Σ3{111} twin boundaries were conducted to have a better insight of local microstructure, and their microstructure would be compared with those in general high-angle grain boundaries.

**Figure 2:**
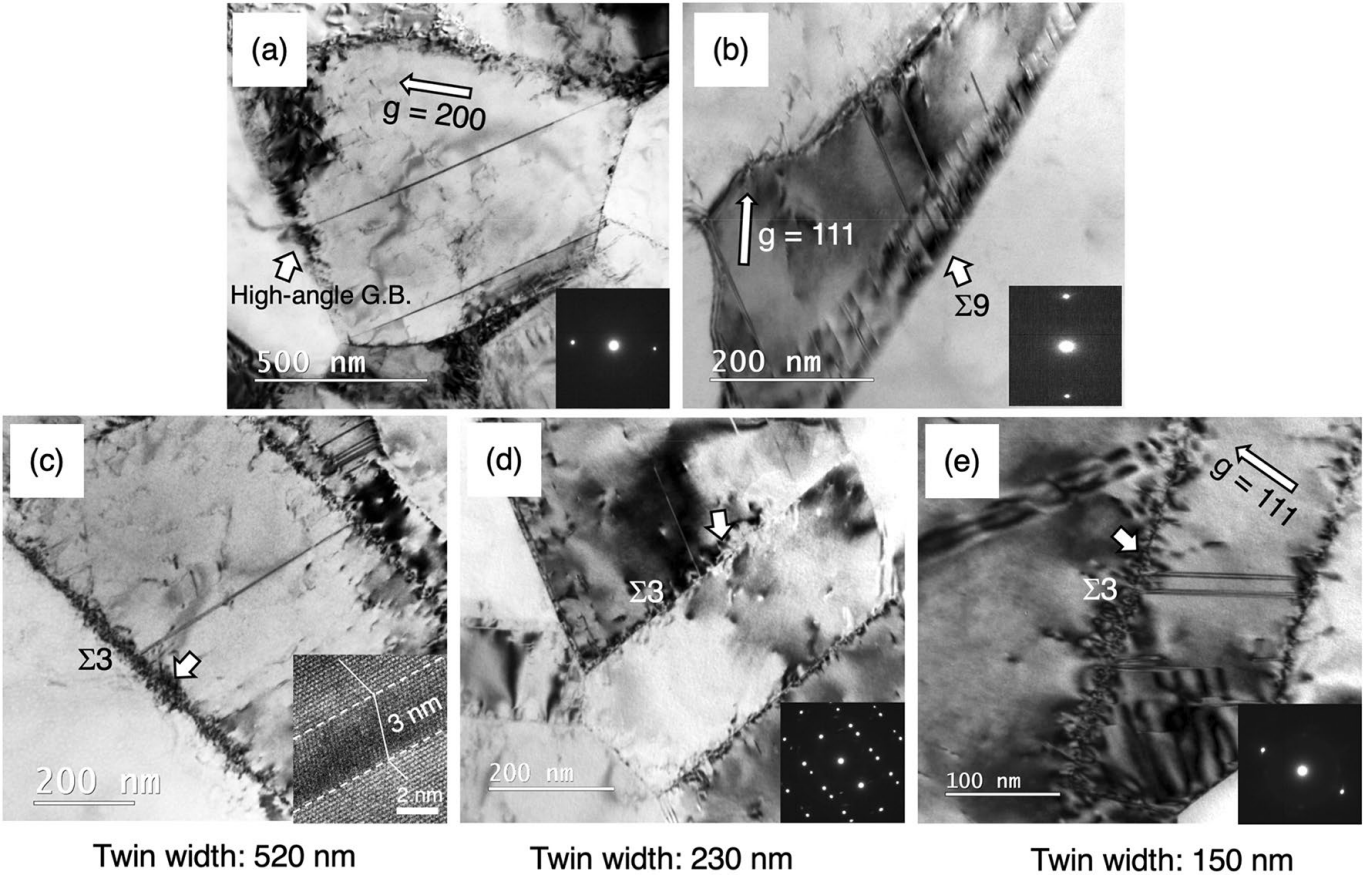
TEM bright field (BF) images show the formation of deformation twins in the sample deformed to the engineering strain = 0.062. (**a**) A deformation twin was nucleated from a general high-angle grain boundary, taken in a two-beam condition with the operative reflection = g_200._ The dark strain contrast along the grain boundary is indicated by a white arrow. (**b**) A [011] Σ9 tilt boundary decorated with a group of grain-boundary dislocations is indicated by a white arrow, where deformation twins were nucleated. The BF image were taken in a two-beam condition with the operative reflection = g_111_. (**c**–**e**) three Σ3{111} twin boundaries (Σ3) having different twin width (520 nm, 230 nm, and 150 nm) were acting as the nucleation sites for deformation twins. Dark strain contrast was uniformly spread along the Σ3{111} twin boundaries.

In Fig. 2, the sample deformed to the engineering strain of 0.062 was characterized by TEM with a particular focus on the deformed regions near grain boundaries. Two general high-angle grain boundaries where the adjoining lattices are tilted by 47.5° along [1 4 10] axis and a [011] Σ 9 tilt boundary are shown in Fig. 2a and b, respectively. As a molecular dynamics simulation reveals that the latter boundary has a great potential to emit partial dislocations^31^, and in fact, both boundaries were found to act as a nucleation site for deformation twin. The dark contrast indicated by the white arrows appears to be induced by a dislocation reaction within the grain boundary, i.e., a deformation twinning event associated with a grain-boundary dislocation dissociation. In Fig. 2c–e, three twin lamellas having different sizes ranging from 100 to 500 nm were observed. The narrow twin width, i.e., nanometer grain size, made the activation of in-grain dislocations difficult, meanwhile deformation twin nucleation at Σ3{111} twin boundaries was evident. Strain contrast that uniformly spread along the Σ3{111} twin boundaries was observed regardless of the width of twin lamellar. This characteristic localized strain contrast within the Σ3{111} twin boundaries as well as deformation twins observed near/at twin boundaries shown in the three TEM images were not identified by the EBSD analysis possibly because of the limitation of EBSD’s spatial resolution, i.e., the size of deformation twins (~ 3 nm) and the extremely localized strain within Σ3{111} twin boundary are too small to be identified.

**Figure 3:**
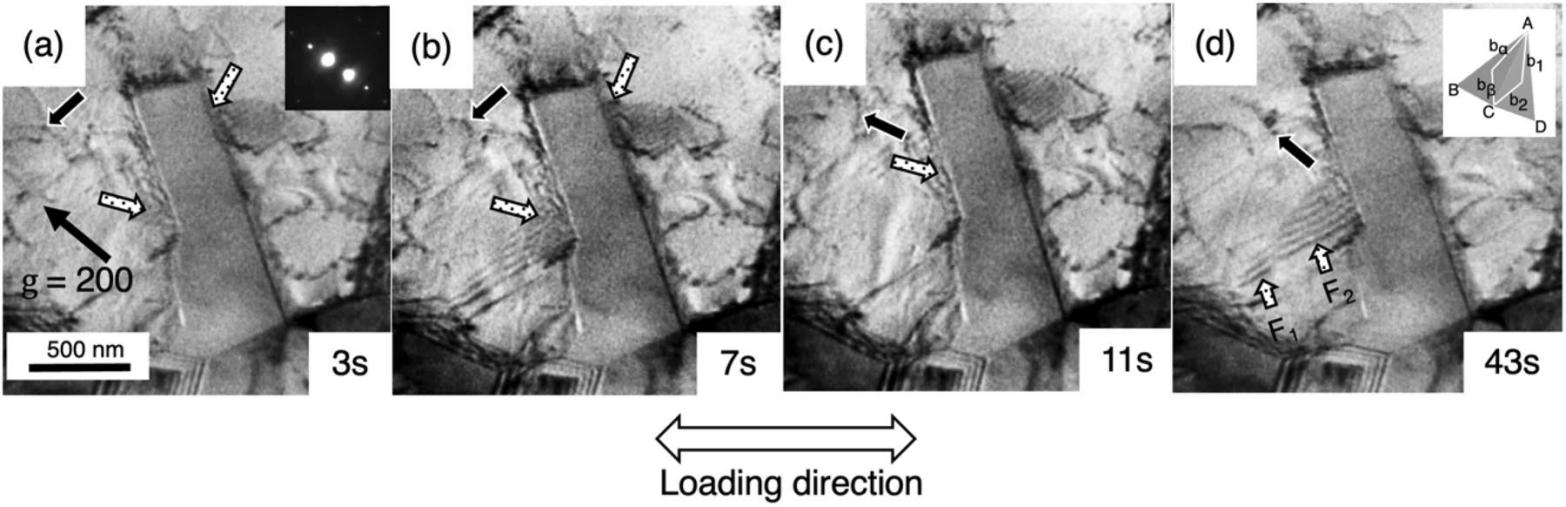
Selected frames of a TEM in-situ deformation test video data showing an area near the Σ3{111} twin boundary. The video (see provided Supplementary Video) was recorded in a two-beam condition with the operative reflection = g_200_. (**a**) The initial stage of a stacking fault emission event. The arrows filled with dots indicates the stacking faults nucleated from the boundaries. (**b**–**d**) the continuous emission of stacking faults from the Σ3{111} twin boundary. The periodic contrast change was observed during the deformation and may be the result of overlapping of stacking faults.

Figure 3a–d are selected frames extracted from an in-situ TEM tensile deformation test movie (full video provided in Supplementary Information). Several stacking faults were nucleated on both sides of an annealing twin grain, i.e., the two Σ3{111} twin boundaries. In addition, a few dislocations were observed in the upper left corner. These are indicated by the arrows filled with dots and the black arrow in the figures, respectively. It is worth noting that the stacking faults were inclined relative to the incident electron beam in this imaging condition thus they look wider. In the initial stage of plastic deformation, the nucleation of stacking faults from both sides of an annealing twin grain, as well as a few dislocations gliding took place simultaneously as seen in Fig. 3a and b. The observed stacking faults emission from the Σ3{111} twin boundary without having noticeable in-grain dislocation − Σ3{111} twin boundary interaction suggests that no localized stress concentration field was generated by the dislocation pile-up for twin nucleation. The contrast of the stacking fault on the left hand side of Σ3{111} twin boundary was vanished in Fig. 3c, which could be ascribed by the emission of the trailing partial dislocation or the third overlapping stacking fault that made the contrast of the stacking fault nearly invisible, i.e., phase angle α becomes 2π as equivalent as the perfect lattice^32^. The leading Shockley partial dislocations were continuously emitted from the Σ3{111} twin boundary (Fig. 3d) as the in-grain slip was significantly suppressed and the stacking fault became visible again. The outer fringe contrast of the fault 2 (F_2_) turned from dark to bright, i.e., two stacking faults (F_1_ and F_2_) were overlapping and showed the reversal fringe contrast. These spontaneously successive emission events occur rapidly at the Σ3{111} twin boundary and is found to be characteristically different from the one induced by in-grain dislocations− Σ3{111} twin boundary interaction in the CG counterpart in Supplementary Fig. S1^26^. A Thompson tetrahedron inserted in Fig. 3d provides the crystallographic relationship between the Σ3{111} twin boundary and the emitted stacking fault. In the deformation process, Shockley partial dislocations having the Burgers vector of b_1_ and b_2_ will glide on the twin plane of $\overline{\text{ACD}}$ while Shockley partial dislocations having the Burgers vector of ${\text{b}}_{\alpha }$ and ${\text{b}}_{\beta}$ on the plane of $\overline{\text{ABC}}$ will be emitted to the grain.

**Figure 4:**
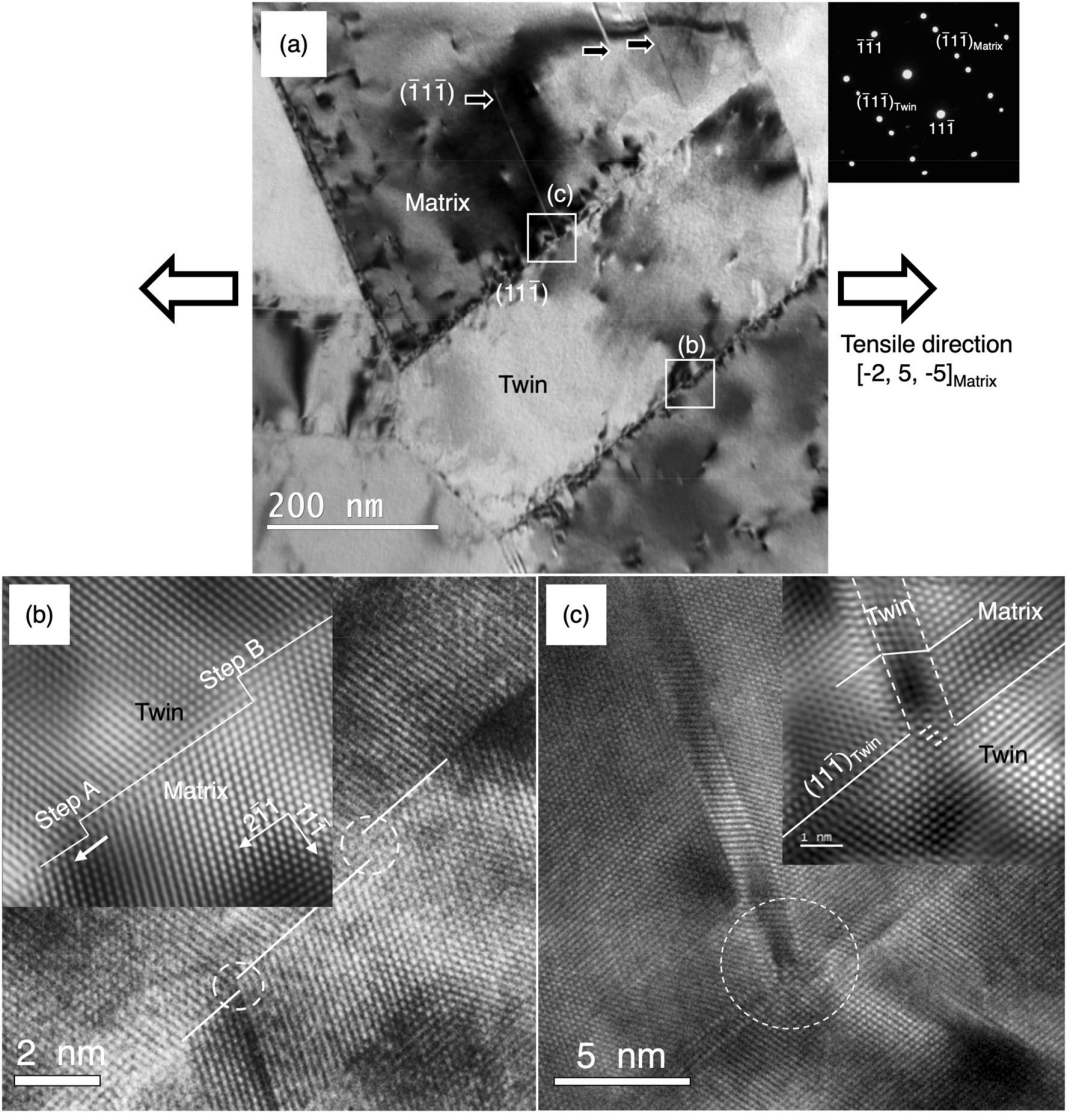
(**a**) BF TEM image taken in a zone axis = [011] shows the deformed microstructure and deformation twins at a Σ3{111} twin boundary. The Σ3{111} twin plane and deformation twin plane are designated to be (11$\overline{1}$)_matrix_ and ($\overline{1}$1$\overline{1}$)_matrix_ based on the attached inset of diffraction pattern. The tensile direction indicated by double white arrow is estimated to be [$\overline{2}$5$\overline{5}$]_matrix_. (**b**) HRTEM image shows two atomistic disconnections, circled by white-dotted line. The inset Fourier-filtered HRTEM image corresponding to the white-dotted lines shows two disconnections (A and B). (**c**) HRTEM image shows a deformation twin nucleated from a disconnection having 3-monolayers height at the Σ3{111} twin boundary. The inset Fourier-filtered HRTEM image corresponding to the nucleation site circled by white-dotted line shows the detailed atomic structure.

A BF image in Fig. 4a illustrates a pair of deformed Σ3{111} twin boundaries and a few thin deformation twins (black arrow). The upper side Σ3{111} twin plane and the leftmost deformation twin plane were identified to be (11$\overline{1}$) and ($\overline{1}{\text{1}}\overline{1}$), respectively, from the selected area electron diffraction (SAED) pattern taken from a [011]_fcc_ zone axis in the inset of Fig. 4a. The tensile axis (a double-white arrow) was determined to be nearly [$\overline{2}$5$\overline{5}$] with respect to the crystal frame of matrix. Applying the Schmid’s law to estimate the resolved shear stresses on slip planes, the [$\overline{2}$5$\overline{5}$]_matrix_ tensile axis causes the resolved shear stress applied on {111} 〈112 〉 leading Shockley partial dislocation to be larger than those applied on {111} 〈110 〉 perfect dislocations and {111} 〈112 〉 trailing Shockley partial dislocations in both Σ3{111} twin plane and deformation twin plane. For example, the Schmid factor for a/6[2 $\overline{1}$1] leading Shockley partial dislocation gliding on (11$\overline{1}\text{)}$ planes is as high as 0.488, i.e., twice larger than that for the a/6[$\overline{1}$21] and the a/6[112] trailing partial dislocations, which would promote the leading Shockley partial dislocation to glide on the planes adjacent to the twin plane during plastic deformation. To probe detailed insights of how the Σ3{111} twin boundary being deformed and the origin of those localized contrasts around Σ3{111} twin boundaries, two squared regions on the Σ3{111} twin boundaries (Fig. 4a) were examined by the high-resolution TEM (HRTEM) imaging technique. Figure 4b and c are the corresponding HRTEM images taken from a [011]_fcc_ zone axis, showing (i) atomistic steps with the height of two or three {111} lattice spacings (Fig. 4b), which are usually described as twinning dislocations with disconnection character^33,34^. We denote them as twin boundary disconnection in here (ii) a deformation twin nucleated at a step (disconnection) (Fig. 4c). A pair of experimental and Fourier-filtered HRTEM images in Fig. 4b exhibit two disconnections (A and B) having a two- and three- monolayer height, respectively, while Fig. 4c shows a deformation twin nucleated from a three-monolayer height step (disconnection). The disconnection consisted of partial dislocations on the successive (11$\overline{1}$) planes causes the Σ3{111} twin boundary to migrate into the neighboring matrix-lamellae thus consequently thickening the original twin-lamellae. In Fig. 4c, the twin boundary disconnection appears to act as a nucleation site for deformation twin. The characteristic strain contrast along the Σ3{111} twin boundaries observed in the BF TEM images appears to be originated from the strain fields associated with disconnections and/or dislocations gliding on the several (11$\overline{1}$) planes adjacent to the Σ3{111} twin plane.

**Figure 5:**
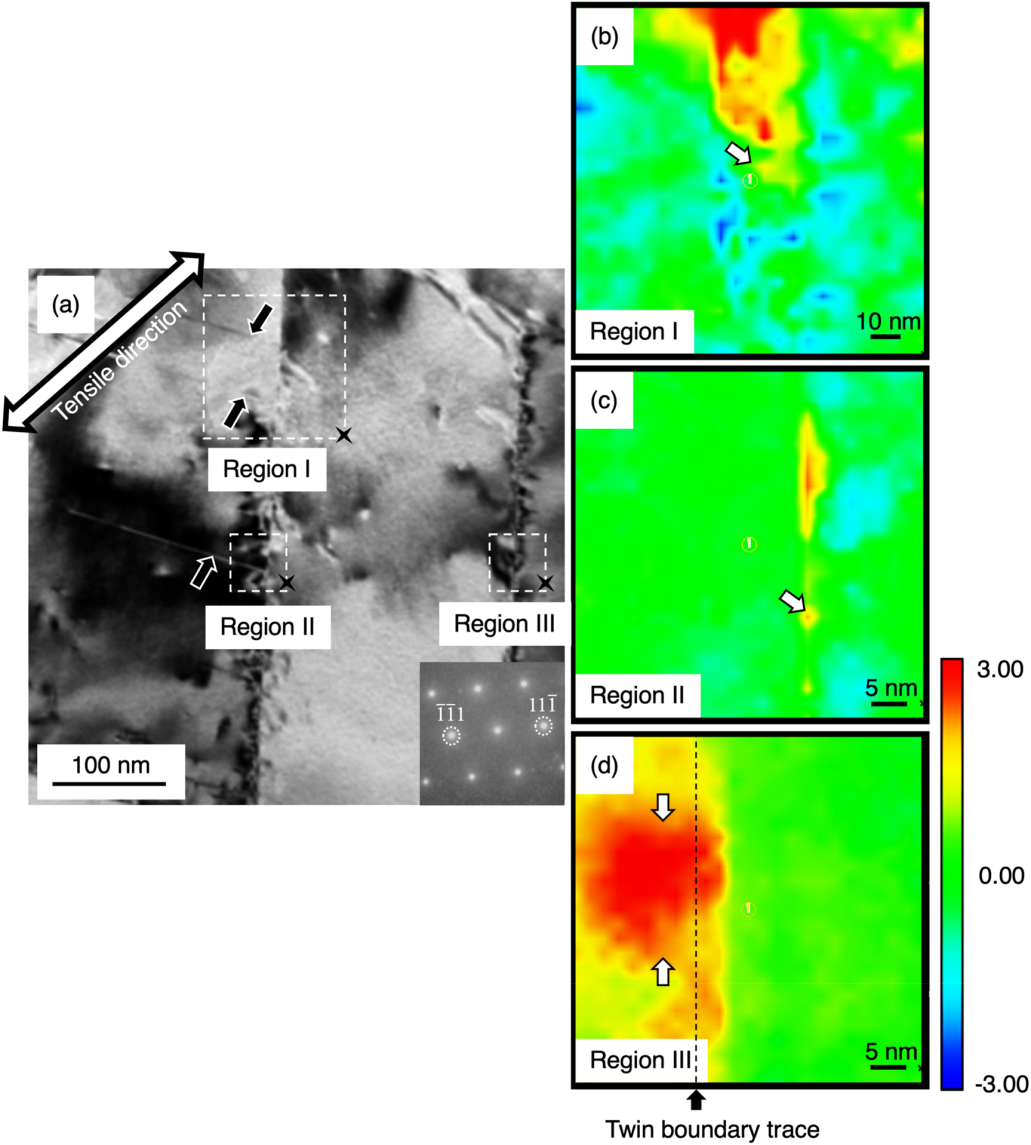
(**a**) BF image shows three deformation twins nucleated from one side of a Σ3{111} twin boundary. Three regions of interest are squared by white dash lines and marked as Region—I, II, and III. (**b**–**d**) Strain maps of the three regions in [11$\overline{1}$] direction show the strain distribution. The color map represents strain (%). Red indicate tensile strain and Blue indicates compression strain.

Strain mapping by the parallel-nanoprobe scanning transmission electron microscopy (μP-STEM) technique was performed on the regions in/near an Σ3{111} twin boundary in Fig. 5. Three regions of interest are marked as Region—I, II, and III. A section of the Σ3{111} twin boundary and deformation twins were included in Region—I and II to compare with the Region-III where deformation twinning has not taken place. First, the Σ3{111} twin boundary was tilted to the edge-on condition, such that the incident electron beam direction was parallel to the Σ3{111} twin boundary plane. A series of over 600 electron diffraction patterns were acquired from the 125 × 125 nm square in Region-I and the 50 × 50 nm square in Region- II and III, with a distance of 5 nm and 2 nm between diffraction patterns, respectively. The probe size was less than 2 nm for this analysis. Secondly, to calculate the strain from the diffraction patterns, a custom-made data processing software package combined with the nanobeam diffraction (NBD) analysis software package (System In Frontier, Inc., Japan) was employed. The software detects the central point of all recognizable diffraction spots in each of electron diffraction patterns, then computes changes in distance between individual diffraction spots and the origin (the center spot). The strain relative to the reference point will be calculated and visualized according to a specific direction. The reference point can arbitrary be selected but usually chose from an unstrained area. In light of distinct strain contrast arising from dislocation reactions on Σ3{111} twin boundary, the relative strain along a [11$\overline{1}$] crystallographic orientation, i.e., in the direction of Σ3{111} twin boundary normal, was calculated in here. Finally, a 2D strain map in a [11$\overline{1}$] crystallographic orientation was generated. The least strained position within each of regions (black cross marker in Region I, II, and III) was selected to be the reference point, thus the positive (tension) and negative (compression) strain displayed in here were relative to the reference point. Figure 5b shows that a huge amount of tensile strain arose in part of the Σ3{111} twin boundary, while the tensile strain below that area of the Σ3{111} twin boundary (white arrow) was much less pronounced. Similarly, another localized tensile strain on the Σ3{111} twin boundary was observed in Fig. 5c drastically decreased only a short distance apart. Based on these strain maps, the areas where the deformation twins nucleated have a relatively less pronounced strain field, suggesting that strain relaxation associated with deformation twinning appears to take place within the Σ3{111} twin boundary, which reduces the stored strain. Region-III in Fig. 5d demonstrates a pronounced localized strain field (white arrows) at the Σ3{111} twin boundary. Based on the corresponding microstructure shown in Fig. 4b, the disconnections (steps) are a high strain site. Some of the disconnections would act as a nucleation site for deformation twin if the deformation could continuously proceed as localized stress exceeding the twinning stress.

## Discussion

The main finding of the present study is that the Σ3{111} twin boundaries in the UFG TWIP steel can be a nucleation site for deformation twins by a possible three-step deformation process: (1) forming disconnections as a result of twin boundary dislocation dynamics under applied stresses, (2) multiplying and propagating disconnections on the originally atomistically smooth boundaries, (3) increasing localized strain at disconnections stimulating preferential deformation twin nucleation. Here, the differences in the microstructural response of the Σ3{111} twin boundaries between the present UFG TWIP steel and the coarse-grained counterpart^26^ will be discussed.

Deformation activities at/near grain boundaries in face-centered cubic metals have been extensively described, both in experimentally^26,35,36^ and theoretically^31,37–44^. Grain boundaries can be an effective dislocation source if their rotation axis is well aligned with the dislocation lines of Shockley partials or perfect dislocations such as a [112] Σ21 tilt boundary^45^ or a [011] Σ9 tilt boundary (Fig. 2b)^31^, or if their excess free volume within grain boundary regions could facilitate the formation of Shockley partial dislocation. On the other hand, there is absence of grain boundary dislocation and free volume in coherent Σ3{111} twin boundary. Accordingly, it is generally believed that the coherent Σ3{111} twin boundary unlikely acts as a proactive dislocation source. To explain the nucleation of deformation twin at Σ3{111} twin boundaries shown in Fig. 2b–d, the coherent Σ3{111} twin boundaries in the present UFG TWIP steel need to be either inherently defective or becoming defective during the plastic deformation.

**Figure 6:**
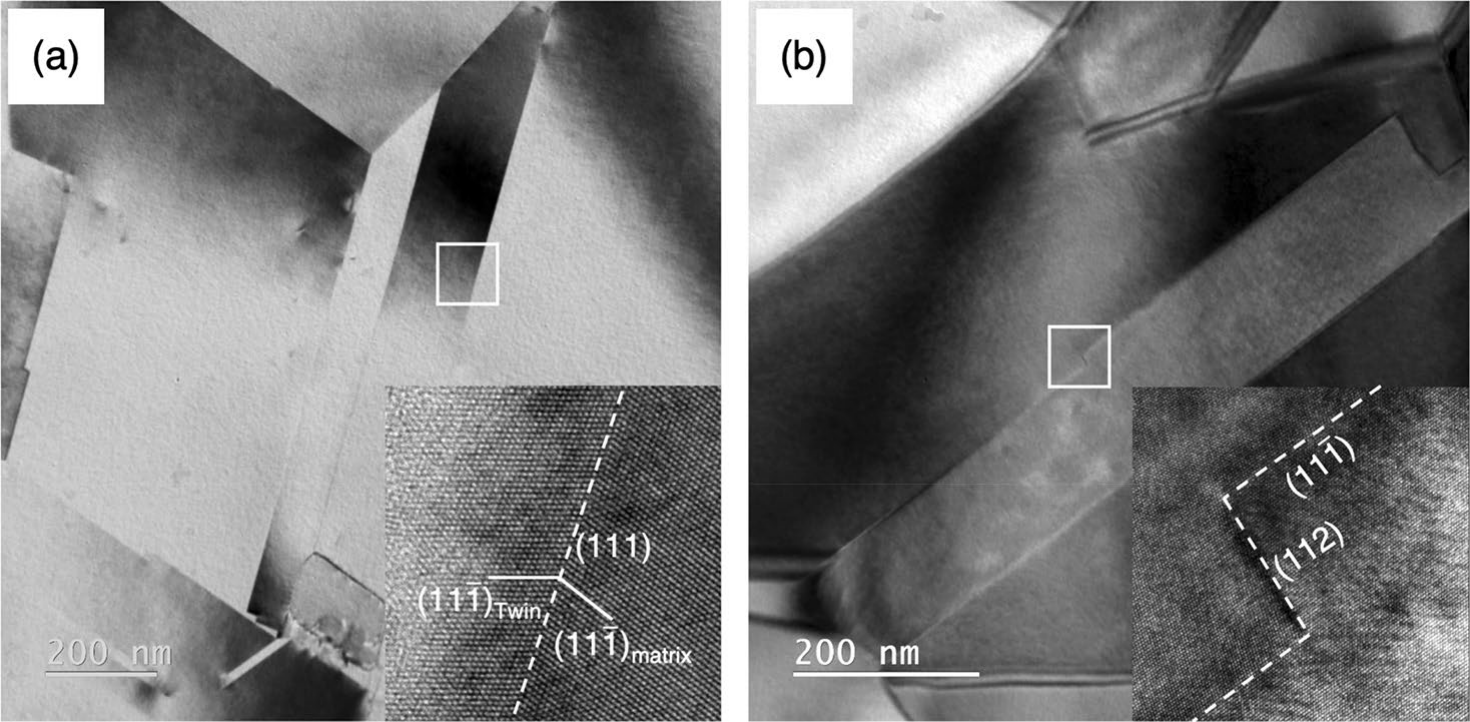
Two sets of BF TEM and HRTEM images show two representative boundary structures of two Σ3{111} twin boundaries in an undeformed sample. (**a**) The atomic structure of the region marked by a white square is an atomistically coherent and flat twin boundary. (**b**) The atomic structure of the region marked by white square represents a 5 nm (112) incoherent twin boundary.

In the present study, the structure of the representative coherent Σ3{111} twin boundaries prior to tensile straining was a perfect and atomistically smooth configuration with no disconnection (defective step), as shown in Fig. 6a. Although some large incoherent Σ3{112} twin boundaries were observed along the coherent Σ3{111} twin boundaries (Fig. 6b), these incoherent Σ3{112} twin boundaries were formed by annealing and considered to be stable compared with the under 1 nm height steps formed by deformation^34,46–48^. The characteristic strain contrast that uniformly spreads along the deformed Σ3{111} twin boundaries (Fig. 2c–e), was not present on the incoherent Σ3{112} twin boundaries, thus the accumulated strain on this type of incoherent twin boundary is not high enough to trigger the deformation twin nucleation, if that is the case.

**Figure 7:**
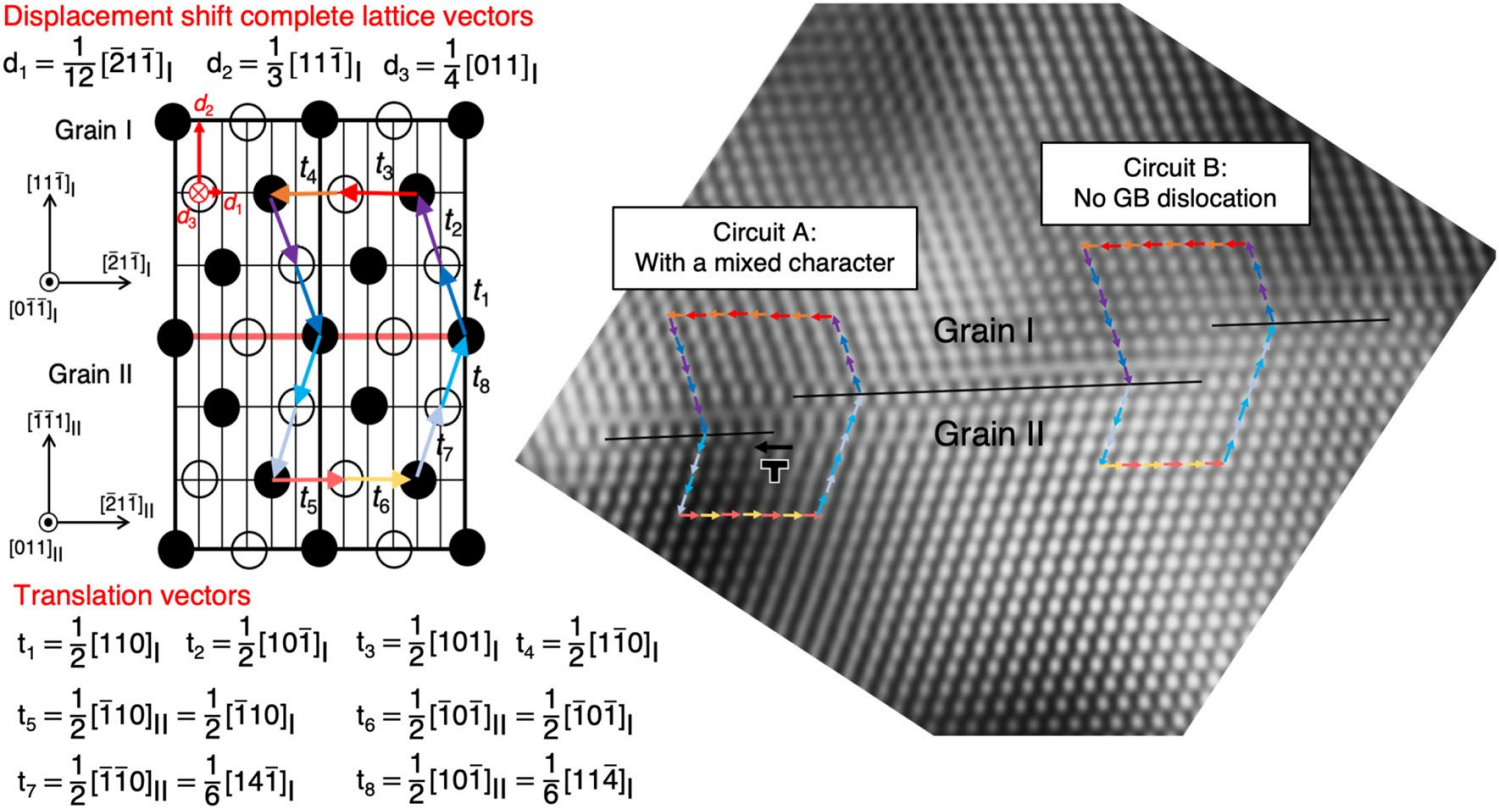
Two closed Burgers circuits labeled by “A” and “B” used to analyze the disconnections imaged in Fig. 4b. The Burgers vector in the circuit A was identified to have a mixed character, while the Burgers vector of dislocation in the circuit B was not identified. The disconnection in the circuit B could be free of dislocation, or there were two partial dislocations having opposite signs. The corresponding lattice vectors are schematically illustrated and described with a proper coordinate matrix transformation, as labeled from t_1_ to t_8_. The corresponding displacement shift complete (DSC) lattice vectors are also specified to be d_1_ = 1/12 [$\overline{2}{\text{1}}\overline{1}$]_I_, d_2_ = 1/3[11$\overline{1}$]_I_, and d_3_ = 1/4[011]_I_.

To determine the Burgers vector of the twin boundary dislocations associated with the twin boundary disconnection, the Burgers circuit approach^34,49,50^ was applied as schematically illustrated in Fig. 7. Two enclosed Burgers circuits labeled “A” and “B” were drawn to analyze the twin boundary disconnections. The lattice vectors traveling around the circuits were recorded and labeled from **t**_1_ to **t**_8_, i.e., the vectors from **t**_1_ to **t**_4_ and the vectors from **t**_5_ to **t**_8_ were defined with respect to the crystal frame of grain I (Twin) and grain II (Matrix), respectively. The Burgers vector of twin boundary dislocation can be determined by the summation of the lattice vectors when lattice vectors of **t**_5_ ~ **t**_8_ were coordinately transformed into the ones in crystal frame of twin. The Burgers vector of the twin boundary dislocation then can be expressed as the following:

1 $${\mathbf{b}}_{{{\text{GB}}}} = - \sum\nolimits_{1}^{8} {t = 1/6[112]_{{\text{I}}} },$$

where the translation vectors of t_1_ ~ t_8_ are listed in Fig. 7. This Burgers vector of the twin boundary dislocation in the circuit A was determined to have a mixed character, i.e., the Burgers vector of twin boundary dislocation can be described by two specific displacement shift complete (DSC) lattice vectors:

2 $${\mathbf{b}}_{{{\text{GB}}}} = 1/6\left[ {112} \right]_{{\text{I}}} = \, - {\text{d}}_{1} + {\text{ d}}_{3} = \, - 1/12[\overline{2}1\overline{1}]_{{\text{I}}} + 1/4[011]_{{\text{I}}},$$

where d_1_ and d_3_ are the DSC lattice vectors illustrated in the schematic of Fig. 7. On the other hand, the summation of the lattice vectors in the circuit B is zero. This result indicates that the three-monolayer height disconnection could be free of lattice defects, or there are two partial dislocations having opposite signs. In the present study, multiple Σ3{111} twin boundaries were carefully examined prior to tensile straining and confirmed to have no disconnection as discussed in Fig. 6. Thus, to the best of the authors knowledge, the three-monolayer height disconnection in here was formed by deformation rather than inherently defective.

A quantitative analysis of nanoscale strain distribution indicates the correlation between local strain level and deformation twinning behavior. The strain level at/near the disconnection in Fig. 5d is approximately 3.65% ± 0.32 in average by taking all individual data points in the near- disconnection region into account, while the strain level near the disconnection with a deformation twin in Fig. 5c is significantly low, approximately − 0.02% ± 0.52 in average. Our Burgers circuit approach indicates that the strain concentration at the step in Fig. 5d is notable due to the contribution of the twin boundary dislocations and this is consistent with the strain field of the Shockley partial dislocation having a pure edge component^51,52^. In contrast, the disappearance of localized strain field in Fig. 5c is likely caused by the nucleation of deformation twin.

**Figure 8:**
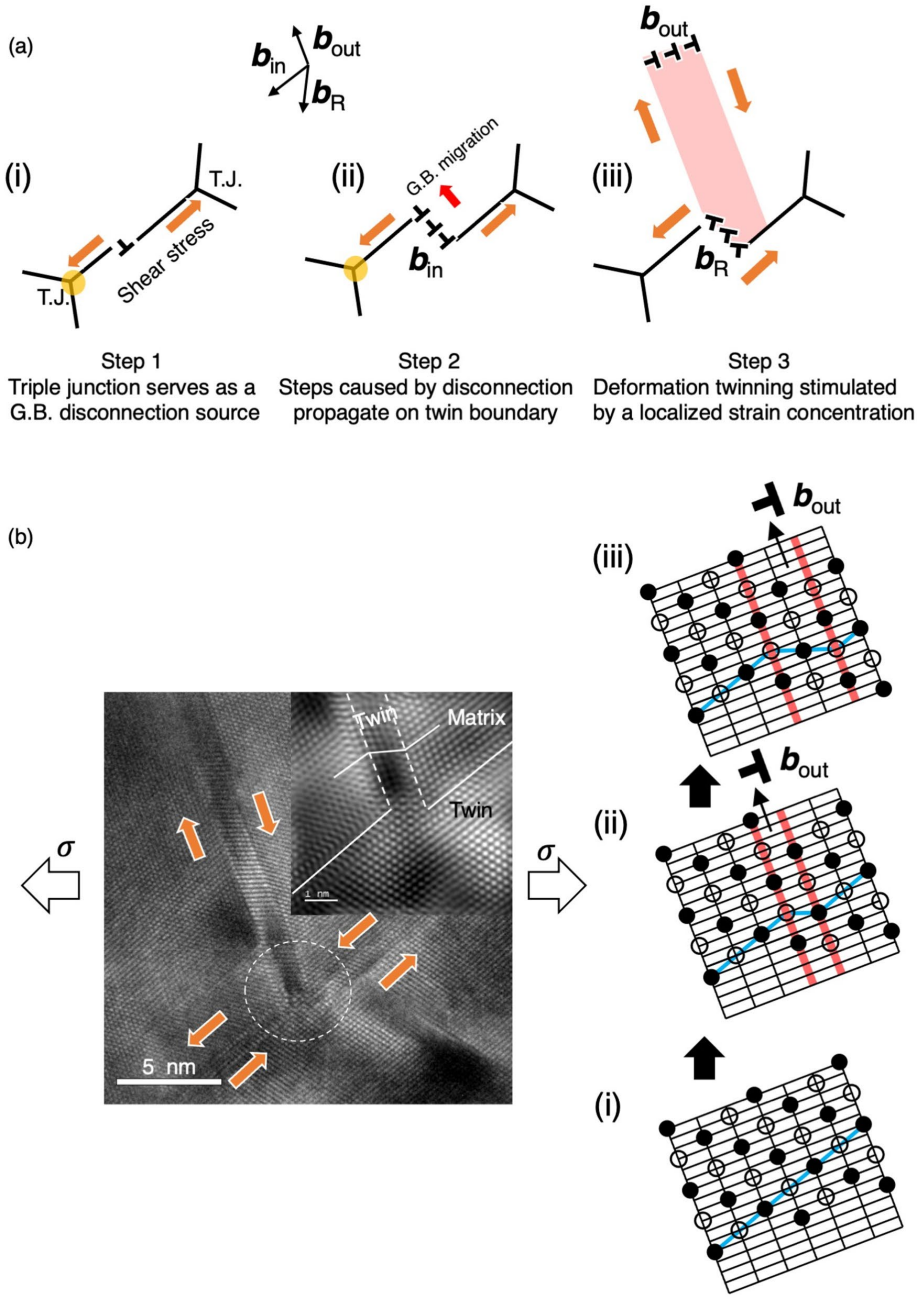
(**a**) A schematic illustration shows a three-step mechanism to form a nucleation site for a deformation twin at an originally coherent Σ3{111} twin boundary. Step 1: Triple junction serving as a source of grain boundary disconnection. Step 2: Steps caused by disconnection propagating on the twin boundary. Step 3: Defective steps containing mixed character twin boundary dislocations generate a localized strain accumulation progressively, thus stimulating the deformation twin nucleation. (**b**) Deformation twinning processes (i to iii) are schematically illustrated. The emission of closely spaced overlapping stacking faults lying on parallel ($\overline{1}$1$\overline{1}$) planes is demonstrated with a step-by-step manner.

The mechanism to form a nucleation site for deformation twins is schematically illustrated in Fig. 8a, which represents the case in Fig. 4c where a 4-atomiclayered deformation twin was formed from a 3-monolayer height disconnection. In general, when grain interior dislocation sources become deplete or inactive due to the grain size constraint, grain boundary sliding would occur and generating dislocations or disconnections mainly at grain boundary triple junctions^27,53,54^. As a result, these disconnection propagations on Σ3{111} twin boundary results multiple disconnections, i.e., the atomistic step having few monolayers in height, on the originally atomistically smooth twin boundaries, that accommodates the misfit between two sides of grains across the twin boundary. The multiple disconnections emitted sequentially from triple junction and coexisted on the neighboring {111} twin plane are similar to the internal behavior observed by in situ atomistic resolution TEM experiment^54^ where a Σ11(113) coherent grain boundary showed a layer-by-layer migration behavior due to the sequential nucleation and lateral motion of grain boundary disconnections. This disconnection activity combination with shear coupled grain boundary migration was also observed in a nanocrystalline gold metal by in situ TEM deformation experiments and considered to be the dominant deformation mechanism^55^. The disconnections can act as preferential nucleation sites for stacking faults because the localized stress concentration field at/near disconnections could exceed the twinning stress during plastic deformation, thus allow in-grain dislocation slip transfer in the later stage of plastic deformation^49,56^. This grain boundary sliding process appears to be phenomenologically similar to the grain boundary migration mechanism originally proposed by Hirth^49,57^ and experimentally observed in the deformed microstructure of several nanograined coppers^46,47,58,59^.

The nucleation mechanism of ~ 1 nm thick deformation twin at a disconnection is schematically illustrated in Fig. 8b. The emission of closely spaced overlapping stacking faults lying on parallel ($\overline{1}$1$\overline{1}$) planes from (i) to (iii). The Burgers vector of residual twin boundary dislocations after the nucleation of deformation twin can be determined by the following relationship:

3 $${\mathbf{b}}_{{\text{R}}} = {\mathbf{b}}_{{{\text{in}}}} {-}{\mathbf{b}}_{{{\text{out}}}},$$

where **b**_R_ is the Burgers vector of residual twin boundary dislocation, **b**_in_ is the Burgers vector of the dislocations within a disconnection, **b**_out_ is the Shockley partial dislocation emitted from the disconnection. Based on the crystallographic orientation relationship between the Σ3{111} twin boundary and deformation twin, **b**_in_ and **b**_out_ can be designated to be 1/6[1$\overline{2}\overline{1}$] and1/6[21$\overline{1}$] respectively. As a result, the magnitude of the residual twin boundary dislocation, i.e., **b**_R_ = 1/6 [$\overline{1}\overline{3}{\text{0}}$], becomes larger than that of **b**_in_ and **b**_out_. This suggests that the formation of deformation twin from a disconnection is unlikely to be an energetically favorable event from the Burgers reaction standpoint. However, since the deformation twin as actually nucleated, the released elastic strain energy by the deformation twin nucleation could be larger than the net dislocation energy increase. By comparing the deformation twin nucleation on Σ3{111} twin boundaries in UFG and CG TWIP steels, it is concluded that localized strain accumulation is essential to stimulate the deformation twin nucleation in both grain sizes. On the other hand, the mechanism to generate the localized strain concentration depends on the grain size, that is, reducing the grain size to under 1 µm alters the region where the dominant dislocation activities occurred from grain interior to grain boundary and also allows energetically less favorable reactions (nonconventional deformation mode) proceed.

## Conclusions

The deformation twin nucleation mechanism on Σ3{111} twin boundaries in a UFG Fe-31Mn-3Si-3Al austenitic TWIP steel was found to be characteristically different from that in the CG counterpart. The detailed microstructural investigation using in-situ TEM deformation, HRTEM and µP-STEM techniques has drawn the following conclusions:

1. The deformation twin nucleation can occur on all type of observed boundaries regardless of the grain boundary misorientation (character), i.e., general high-angle grain boundary, boundaries having a particular tilt axis ([011] Σ9), and low-energy coherent Σ3{111} twin boundaries are all able to be a nucleation site for deformation twins.
2. The formation of twin boundary disconnections appears to be governed by the motion of Shockley partial dislocations on the Σ3{111} twin boundary. These disconnections (defective steps) would accumulate strain and serve as the nucleation sites of deformation twins when the localized stress level exceeds the twinning stress.
3. The mechanistic difference to generate localized strain concentrations on coherent Σ3{111} twin boundaries between the UFG and the CG alloys is concluded to be originated from a unique characteristic of the UFG microstructure. The grain size constraint suppresses the in-grain dislocation activities in the UFG steel and stimulates the twin boundary dislocation activities leading to the twin boundary disconnections which act as the deformation twin nucleation sites. On the other hand, the observed periodic contrast reversal of stacking fault during the deformation twin nucleation at Σ3{111} boundaries indicates that the deformation twin formation process is likely identical between the UFG and CG microstructures once nucleated, partly due to the identical chemistry and SFE.

## Materials and methods

A carbon-free UFG (0.79 ± 0.39 μm) high-manganese austenitic steel TWIP steel with intermediate SFE (40 mJ m^−2^) was fabricated for this work^29^. The chemical composition of the steel was Fe-31Mn-3Al-3Si wt%. The as-received TWIP steel was cold rolled from 12 to 1 mm (92% reduction) by multi-pass cold rolling and then heat treated in a salt bath at 950 °C for 5 min followed by water quenching to obtain the aimed grain size.

Sheet type tensile test pieces with a specific dimension, 13 × 2 mm rectangular shape, were sliced and mechanically thinned to approximately 150 µm thick for deformed microstructure characterization by electron backscatter diffraction (EBSD) and transmission electron microscopy (TEM) techniques. The 150 µm thick sheets were tensile-deformed to 0.046 and 0.062 engineering strain using a testing machine (Kammrath and Weiss Module 200 N) at a strain rate of 4.6 × 10^–4^ s^−1^ at room temperature.

Specimens for post-deformation EBSD analyses were mechanically polished by abrasive paper up to 2000-grit then electropolished. The microstructural features including grain boundary maps and local strain distribution were examined using a TSL OIM EBSD system attached to a FEI Helios 600 dual-beam field emission gun (FEG) scanning electron microscope (SEM) / focused ion beam (FIB) system. EBSD maps were acquired at 30 kV acceleration voltage and 13 mm working distance. The scanning step size was 50 nm. No data clean-up was performed except for the removal of some points with low confidence value.

The local strain distribution was evaluated from the kernel average misorientation (KAM). KAM is a local misorientation defined as the average misorientation of a point with its nearest neighbors in a grain. The average misorientation of a given point is calculated by taking that point as well as all of its nearest neighbors into account with a criterion that the misorientation exceeding threshold 3° will be discarded from the calculation, because these points are considered to belong to the adjacent grains.

Specimens prepared for post-deformation TEM analyses were cut from the center of the deformed sheets. 2 × 2 mm square shape foils were taken then mechanically thinned to 70 μm thick. The final thinning to be partly electron transparent was achieved by using a twin-jet electropolisher (Fischione Model 110) with a 95% acetic acid—5% perchloric acid electrolyte maintained at 17 °C and the applied voltage of 38 V. The TEM characterization was performed using a JEOL JEM 2100 operated at 200 kV with Gatan Orius SC200D and Ultrascan 1000XP cameras.

Specimens for in-situ deformation tests were sliced from undeformed sheets; the shape of the foils, 2 × 2 mm square shape, is identical to the one used for TEM analysis. A square shape foil was fixed on a cartridge-type blade for SATO Holder Duo (Mel-Build Co.)^60^ after thinned to be partly electron transparent. The strain rate was controlled at approximately 6.7 × 10^–5^ s^−1^ in this study. All videos were recorded in the bright field (BF) mode and a FEI Titan 80–300 S/TEM operated at 300 kV was used. Gatan Orius SC200D camera and Digital Micrograph with the high-resolution streaming video plug-in were used for video recording.

The FEI Titan 80-300 V S/TEM also was used in this analysis. Strain mapping was performed using the parallel-nanoprobe scanning transmission electron microscopy (µP-STEM) technique. The µP-STEM employed a 10 µm second condenser aperture to reduce the beam semi-convergence angle to be 0.13 mrad and obtained less than 2 nm probe size.

## Supplementary Information

Supplementary Information 1.

Supplementary Video 1.
