# Supplementary material for: Investigating the dislocation reactions on Σ3{111} twin boundary during deformation twin nucleation process in an ultrafine-grained high-manganese steel
Source: Sci Rep. 2021 Sep 29;11:19298. doi: 10.1038/s41598-021-98875-z (PMC8481298; doi:10.1038/s41598-021-98875-z)
Supplement: Supplementary file 1 — Supplementary Information 1. [file 41598_2021_98875_MOESM1_ESM.docx]

**Investigating the dislocation reactions on Σ3{111} twin boundary during deformation twin nucleation process in an ultrafine-grained high-manganese steel**

Chang-Yu Hung^a,^*, Tomotsugu Shimokawa^b^, Yu Bai^c,f^, Nobuhiro Tsuji^c,d^,

Mitsuhiro Murayama^a,e^*

^a^ Department of Materials Science and Engineering, Virginia Tech, Blacksburg, VA 24061, USA

^b^ Faculty of Mechanical Engineering, Kanazawa University, Kanazawa, Ishikawa, 920-1192, Japan

^c^ Department of Materials Science and Engineering, Kyoto University, Yoshida-honmachi, Sakyo-ku, Kyoto 606-8501, Japan

^d^ Elements Strategy Initiative for Structural Materials, Kyoto University, Yoshida-honmachi, Sakyo-ku, Kyoto 606-8501, Japan

^e^ Institute for Materials Chemistry and Engineering, Kyushu University, Kasuga, Fukuoka 816-8580, Japan

^f^ current address: School of Materials Science and Engineering, Dalian University of Technology, Dalian 116024, China

***Corresponding authors**

Chang-Yu Hung ([changyu1@vt.edu](mailto:changyu1@vt.edu)) TEL: 540-231-0466, FAX 540-231-1963

Mitsuhiro Murayama ([murayama@vt.edu](mailto:murayama@vt.edu)) TEL: 540-231-9470, FAX 540-231-1963

**Contact information of authors**

Chang-Yu Hung E-mail: changyu1@vt.edu

Tomotsugu Shimokawa E-mail: simokawa@se.kanazawa-u.ac.jp

Yu Bai, E-mail: bai.yu.6m@kyoto-u.ac.jp

Nobuhiro Tsuji E-mail: nobuhiro-tsuji@mtl.kyoto-u.ac.jp

Mitsuhiro Murayama E-mail: murayama@vt.edu

**Supplemental Video** A TEM in-situ deformation test video data showing an area near the Σ3{111} twin boundary. This video was recorded in a two-beam condition with operative reflection = g_200_.


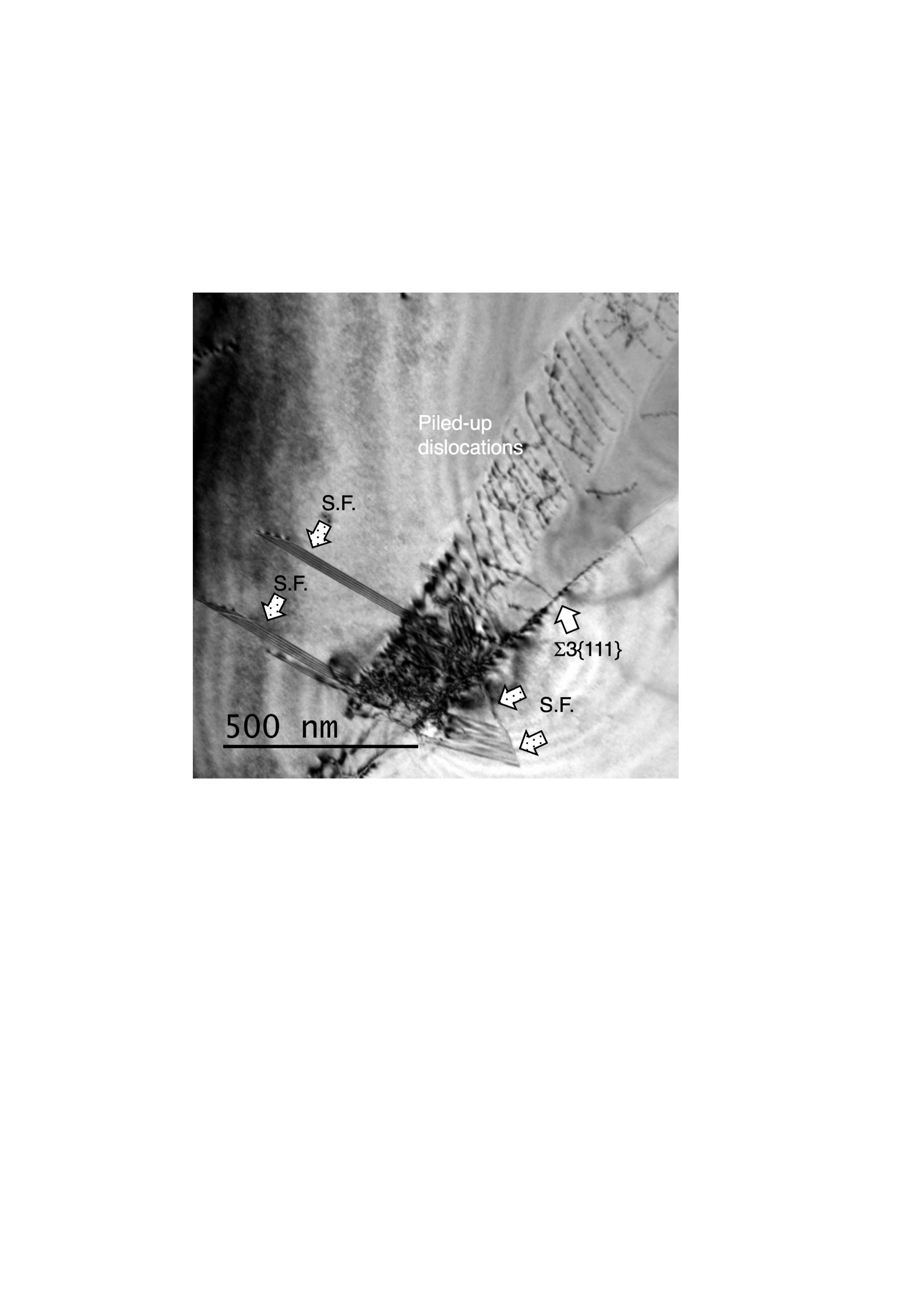


**Fig. S1** Bright field image shows that the formation of stacking faults (S.F.) is induced by the interaction between piled-up dislocations and a Σ3{111} twin boundary in a conventional coarse-grained Fe-31Mn-3Al-3Si austenitic steel. The Stacking faults are indicated by the arrows filled with dots. Σ3{111} twin boundary is indicated by a white arrow.
